# Clinical Significance of Long Non-Coding RNA *CASC8* rs10505477 Polymorphism in Lung Cancer Susceptibility, Platinum-Based Chemotherapy Response, and Toxicity

**DOI:** 10.3390/ijerph13060545

**Published:** 2016-05-30

**Authors:** Lei Hu, Shu-Hui Chen, Qiao-Li Lv, Bao Sun, Qiang Qu, Chong-Zhen Qin, Lan Fan, Ying Guo, Lin Cheng, Hong-Hao Zhou

**Affiliations:** 1 Department of Clinical Pharmacology, Xiangya Hospital, Central South University, Changsha 410008, China; hu773589905@163.com (L.H.); lvqiaoli2008@126.com (Q.-L.L.); scy_csu2016@163.com (B.S.); fanlan_038038@163.com (L.F.); guoying881212@csu.edu.cn (Y.G.); 2 Institute of Clinical Pharmacology, Hunan Key Laboratory of Pharmacogenetics, Central South University, Changsha 410078, China; 3 Department of Oncology, Changsha Central Hospital, Changsha 410006, China; chenshuhui2008@126.com; 4 Department of Pharmacy, Xiangya Hospital, Central South University, Changsha 410008, China; quqiang1983@sina.com; 5 Department of Pharmacy, The First Affiliated Hospital of Zhengzhou University, Central South University, Zhengzhou 450052, China; qcz0378@126.com; 6 State Key Laboratory of Ophthalmology, Zhongshan Ophthalmic Center, Sun Yat-Sen University, Guangzhou 510060, China; kjade.cheng@hotmail.com

**Keywords:** lncRNA CASC8, rs10505477, lung cancer, susceptibility, platinum-based chemotherapy

## Abstract

Long non-coding RNA (lncRNA) *CASC8* rs10505477 polymorphism has been identified to be related to risk of many kinds of cancers, such as colorectal cancer, gastric cancer, and invasive ovarian cancer, and it may be involved in the prognosis of gastric cancer patients who have received platinum-based chemotherapy after surgical treatment. So far, there is no study investigating the clinical significance of lncRNA *CASC8* rs10505477 in lung cancer susceptibility and treatment. In this study, we genotyped 498 lung cancer patients and 213 healthy control subjects to explore the correlation between the rs10505477 polymorphism and lung cancer risk in a Chinese population. Among the 498 patients, 467 were selected for the chemotherapy response and toxicity study. We found that the single nucleotide polymorphisms (SNP) rs10505477 was greatly related to lung cancer risk in male and adenocarcinoma subgroups in recessive model (adjusted OR = 0.51, 95%CI = 0.29–0.90, *p* = 0.02; adjusted OR = 0.52, 95%CI = 0.30–0.89, *p* = 0.02, respectively). It was also closely correlated with platinum-based chemotherapy response in dominant model (adjusted OR = 1.58, 95%CI = 1.05–2.39, *p* = 0.03). Additionally, we observed that *CASC8* rs10505477 polymorphism was significantly relevant to severe hematologic toxicity in non-small-cell lung cancer (NSCLC) subgroup in dominant model (adjusted OR = 0.59, 95%CI = 0.35–0.98, *p* = 0.04) and in additive model (adjusted OR = 0.62, 95%CI = 0.43–0.90, *p* = 0.01). Furthermore, it was found that rs10505477 polymorphism was greatly associated with gastrointestinal toxicity in SCLC and cisplatin subgroups in dominant model (adjusted OR = 7.82, 95%CI = 1.36–45.07, *p* = 0.02; adjusted OR = 1.94, 95%CI = 1.07–3.53, *p* = 0.03, respectively). Thus, lncRNA *CASC8* rs10505477 could serve as a possible risk marker for diagnosing lung cancer, and could be used to forecast the response and toxicity of platinum-based treatment in lung cancer patients.

## 1. Introduction

Lung cancer, containing two primary subtypes: non-small-cell lung cancer (NSCLC) and small-cell lung cancer (SCLC), is a major cause of cancer-related death globally, with an extremely poor overall survival rate [1]. NSCLC, accounting for the most of lung cancers, approximately 85%, could be further classified as squamous-cell carcinoma (SCC), adenocarcinoma (ADC), and large-cell carcinoma [2]. Although cigarette smoking is a well-known major risk factor, genetic factors are also reported to be associated with lung cancer susceptibility [3,4,5]. Platinum-based cytotoxic chemotherapy, one of the various treatment options in clinic, is the standard first-line treatment for lung cancer. However, low objective response rate (ORR) or serious toxicity reaction in some lung cancer patients is often observed in clinic. In brief, late diagnosis, chemoresistance, and toxicity are considered to be major factors that contributed to the poor outcome of lung cancer patients. Therefore, it is urgent to seek out new biomarkers that can accurately predetermine the susceptibility, chemotherapy response, and/or toxicity for lung cancer patients.

So far, a great number of genes have been found to be related to lung carcinogenesis [6,7], efficacy, or toxicity of platinum-based chemotherapy [8,9,10,11]. Although focusing on known genes might yield further understanding in progress and therapy of lung cancer, newly-developed markers such as long non-coding RNAs (lncRNAs) may lead novel insight into the mechanism of lung cancer risk or therapy.

LncRNAs are often considered to be a kind of non-coding RNA over 200 bases with no protein-coding funtion. lncRNAs aroused people’s widespread interest since their transcriptional function were first identified in mouse by Okazaki *et al.* [12]. There has been a growing body of research demonstrating that lncRNAs can participate in gene expression regulation at different levels including modification of chromatin [13], transcription, and post-transcriptional processing [14], involving in various aspects of biological processes, having many kinds of functions in different physiological and pathological processes, and playing vital roles in tumorigenesis and drug efficacy [2,15]. Currently, a number of dysregulated lncRNAs have been identified in various cancers, including lung carcinoma [16,17,18,19]. The aberrant expression of lncRNAs may have important significance in the diagnosis and therapy of cancers, especially lung cancer.

Single nucleotide polymorphisms (SNP) could significantly affect gene expression and function. Increasing evidence demonstrates that SNPs in some lncRNAs are related to tumorigenesis and chemotherapy response [2,20,21]. Gong *et al.*, found that *HOTTIP* rs1859168 or rs5883064, *H19* rs2107425, and *CCAT2* rs6983267 showed strong relationship with risk of lung cancer, *MALAT1* rs619586, *H19* rs2107425 or rs2839698, *CCAT2* rs6983267, *HOTAIR* rs1899663, or rs7958904 and *ANRIL* rs10120688 or rs1333049 were associated with platinum-based chemotherapy response for lung cancer patients [2]. Cancer susceptibility candidate 8 (*CASC8*) gene, a long non-coding RNA, is located in the region of 8q24, which is a gene desert region with no ability of protein coding [22]. Recent evidence suggested that *CASC8* gene polymorphism, such as rs7837328, rs7014346, rs6983267, play important roles in various cancers including prostate cancer [23], breast cancer, colorectal cancer (CRC), and gastric cancer [22]. The SNP rs10505477, located in the intron of lncRNA *CASC8*, showed strong association with the risk of CRC [24,25,26] and the prognosis of gastric cancer [22]. However, to our best knowledge, the implication of lncRNA *CASC8* rs10505477 in lung cancer remains unclear. In present study, we evaluated the relationship of *CASC8* rs10505477 to lung cancer susceptibility, platinum-based chemotherapy response, and toxicity.

## 2. Subjects and Methods

### 2.1. Subjects

The ethics committee of Xiangya School of Medicine of Central South University (Changsha, China) approved this case-control study (registration CTXY-1100082 and CTXY-110008-3). Four-hundred and ninety-eight lung cancer patients with histologically or cytologically confirmed primary lung cancer were recruited from Xiangya Hospital and the Affiliated Cancer Hospital of Central South University from November 2011 and May 2013. Two-hundred thirteen healthy controls were from health examination center of Xiangya Hospital of Central South University. All of the subjects were unrelated ethnic Chinese and provided written informed consent.

The patients that had leptomeningeal metastases or symptomatic brain, active infection, lactation, pregnancy, and other malignant tumors were ruled out from this study. Thus, 467 lung cancer patients were selected for the chemotherapy response and toxicity investigation, satisfying the following conditions: (1) biological therapy and/or radiotherapy were not given before and during chemical therapy; (2) received at least two cycles of chemotherapy treatment; (3) encountered complete follow-up after drug administration; and (4) same methods of imaging were conducted to assess the tumors before and during platinum-based treatment. The platinum-based chemotherapy regimens involved for these selected patients including: platinum with gemcitabine (GP), etoposide (EP), pemetrexed (PP), docetaxel (DP), and paclitaxel (TP), and other platinum-based regimens (platinum with irinotecan or navelbine).

### 2.2. Data Collection

Demographic data and information including sex, age, race, and tumor, node, metastasis (TNM) stages were collected. ORR were estimated on the basis of guidelines as described in Response Evaluation Criteria in Solid Tumors (RECIST) [27]. For data analysis in our study, responders were defined as partial response (PR) and complete response (CR), while non-responders as progressive disease (PD), and stable disease (SD). Toxicities, those related to platinum-based chemotherapy, including gastrointestinal toxicity (diarrhea, nausea and vomiting) and hematologic toxicity (thrombocytopenia, anemia, leucopenia and neutropenia) were evaluated according to the National Cancer Institute Common Toxicity Criteria Version 3.0 (http://ctep.cancer.gov). Severe overall toxicity required the presence of grade 3 or 4 gastrointestinal or hematologic toxicity.

### 2.3. DNA Extraction and Genotyping

Genomic DNA was extracted from the peripheral blood of the subjects using Genomic DNA Purification Kit (Promega, Madison, WI, USA) or FlexiGene DNA Kit (Qiagen, Hilden, Germany) according to the manufacturer’s directions. The DNA was stored at −20 °C until use. The SNP genotyping analysis was performed using the Sequenom MassARRAY system (Sequenom, San Diego, CA, USA). For quality control, DNA sequencing on a random 5% of all samples was conducted to confirm the genotypes, the reproducibility was 100%.

### 2.4. Statistical Analysis

A χ-square test was conducted to evaluate the differences in the distributions of demographic characteristics between lung cancer cases and controls. The goodness-of-fit χ-square test was performed to estimate the Hardy-Weinberg equilibrium for controls. Bivariate logistical regression analysis was used to determine odd ratio (OR), 95% confidence interval (95%CI), and the corresponding *p* value for lung cancer risk analysis, with age and gender as covariates. Bivariate logistical regression analysis was also performed to calculate OR, 95%CI and *p* value of the association of *CASC8* rs10505477 polymorphism with chemotherapy response and toxicity outcomes with sex, age, smoking status, stage, histological type, and chemotherapy regimens adjusted. *p* values that less than 0.05 were used for statistical significance. All data were analyzed using PASW Statistics v18.0 (IBM Co., Armonk, NY, USA) and PLINK 1.9 [28].

## 3. Results

### 3.1. Characteristics of Subjects

Four-hundred ninety-eight lung cancer patients (189 with squamous cell carcinoma, 217 with adenocarcinoma, 69 with small cell carcinoma, and 23 with others) and 213 healthy control subjects were recruited in our study. Table 1 provides a summary of the demographic characteristics distributions of subjects included in our study. Among the 498 cases, 467 were selected for chemotherapy response and toxicity analysis, and the clinical features were summarized in Table 2. The call rate of the SNP rs10505477 is 97.61%. The frequency of *CASC8* rs10505477 G allele (43.49%, 45.95% in cases and controls, respectively) is highly similar to that recorded by NCBI, which reported a minor allele frequency (MAF) of 42.31%. Among controls, the observed genotype distributions of *CASC8* rs10505477 were in Hardy-Weinberg equilibrium (HWE) (*p* = 0.68).

### 3.2. Relationship between CASC8 rs10505477 Polymorphism and Lung Cancer Risk

The relationship between lncRNA *CASC8* rs10505477 polymorphism and lung cancer susceptibility was determined after sex and age adjusted. As shown in Table 3, *CASC8* rs10505477 failed to be statistically related to lung cancer risk. However, the results of stratified analysis demonstrated that the SNP rs10505477 was significantly correlated with risk of lung cancer in male and ADC subgroups in recessive model (adjusted OR = 0.51, 95%CI = 0.29–0.90, *p* = 0.02; adjusted OR = 0.52, 95%CI = 0.30–0.89, *p* = 0.02, respectively, Table 4).

### 3.3. Association between CASC8 rs10505477 Polymorphism and Chemotherapy Response in Lung Cancers

Of the 467 selected patients, 184 were recognized as responders, while 283 as non-responders. After sex, age, histological type, smoking status, stage, and chemotherapy regimens adjusted, it was observed that *CASC8* rs10505477 was greatly correlated with platinum-based chemotherapy response in dominant model (adjusted OR = 1.58, 95%CI = 1.05–2.39, *p* = 0.03, Table 5). No association between rs10505477 polymorphism and chemotherapy response was observed after stratification analysis (Table 6).

### 3.4. Relationship between CASC8 rs10505477 Polymorphism and Hematologic, Gastrointestinal, or Overall Serious Toxicity Induced by Platinum-Based Chemotherapy in Lung Cancers

After sex, age, histological type, smoking status, stage, and chemotherapy regimens adjusted, we observed that *CASC8* rs10505477 was not related to hematologic (Table 7), gastrointestinal (Table 8), or overall severe toxicity (Table 9) of platinum-based chemotherapy. Stratified analysis was performed to further assess the association of *CASC8* rs10505477 with the toxicity induced by platinum-based chemotherapy. We found that *CASC8* rs10505477 polymorphism was statistically correlated with severe hematologic toxicity in NSCLC subgroup in dominant and additive models (adjusted OR = 0.59, 95%CI = 0.35–0.98, *p* = 0.04 in dominant model; adjusted OR = 0.62, 95%CI = 0.43–0.90, *p* = 0.01 in additive model, Table 10). Additionally, it was observed that *CASC8* rs10505477 polymorphism was related to gastrointestinal toxicity in SCLC and cisplatin subgroups in dominant model (adjusted OR = 7.82, 95%CI = 1.36–45.07, *p* = 0.02; adjusted OR = 1.94, 95%CI = 1.07–3.53, *p* = 0.03, respectively, Table 11). No relationship between *CASC8* rs10505477 and overall severe toxicity was discovered when we performed a subtype analysis (Table 12).

## 4. Discussion

It was reported that the SNP rs10505477 in *CASC8* gene is related to risk of several solid tumor malignancies, such as CRC [29,30], gastric cancer [22,31] and invasive ovarian cancer [32]. Study by Sen *et al.*, demonstrated that rs10505477 might be involved in the prognosis of gastric cancer patients receving cisplatin-based chemotherapy after surgical treatment [20]. However, there is no study investigating the relationship between the SNP rs10505477 polymorphism and platinum-based chemotherapy toxicity in any cancer. For the first time, our study showed that lncRNA *CASC8* rs10505477 polymorphism was significantly related to lung cancer susceptibility, platinum-based chemotherapy response and toxicity.

The mechanism by which the SNP rs10505477 modify cancer risk is still unclear. Several hypotheses have been put forward. First, rs10505477 is located in the intronic region of lncRNA *CASC8*, Ma *et al.* [22] hypothesized that it may affect the function of CASC8 through changing its folding structures, disrupting the critical regulatory region of *CASC8* and leading to its dysregulated expression. Second, probably, rs10505477 allele could alter the interactions between the *CASC8* and its cognate gene *POU5F1B* (POU class 5 homeobox 1 pseudogene 1), an acknowledged tumor susceptibility gene [33], by regulating the binding of some transcription factors to promoter of *POU5F1B* gene. Third, it was reported that there existed a strong linkage disequilibrium (LD) between the SNP rs10505477 and the SNP rs6983267, which is also located at 8q24 and has been reported to be related to tumorigenesis [2,22,34,35]. Evidence suggests that the GG genotype of rs6983267 was greatly related to increased lung cancer risk in Han Chinese [35]. Many other studies have also demonstrated the significant association between rs6983267 GG genotype and high risk of several other cancers, such as CRC, prostate cancer, and kidney cancer [26,36,37]. Furthermore, it was reported that the SNP rs6983267 was correlated with the increased CRC risk by enhancing the response to Wnt signaling pathway and combination with promoter of *MYC* [38,39,40], which was aberrantly expressed in many kinds of cancers, including lung cancer [41]. The SNP rs6983267 also could interact with β-catenin-TCF4 complex and promotes the tumorigenesis of CRC. Therefore, probably, the rs10505477 locus could indirectly affect the susceptibility of cancer through its connection with the cancer risk-related rs6983267 mutant. However, the possible role of these two variant and their LD on the function of contiguous gene in risk of cancers including lung cancer warrants further investigation.

It has been reported that *CASC8* gene is located at 8q24, a “gene-desert” region, and overlaps *POU5F1B* gene [20,22], which is identified to be highly homologous to *POU5F1* (POU class 5 homeobox 1) gene, also called *OCT3* or *OCT4* [42]. Additionally, Panagopoulos *et al.*, have reported that *POU5F1B* could produce a protein with similar function to POU5F1 [43]. Study by Hosokawa *et al.*, demonstrated that OCT-3/4 expression was well related to ATP binding cassette transporter G2 (ABCG2), a drug efflux pump gene, in glioblastoma samples, and OCT-3/4 could strengthen the resistance of chemotherapeutic drugs, such as doxorubicin, carboplatin, and etoposide phosphate, in glioblastoma cell lines by elevating the expression of ABCG2 [44]. Ota *et al.* [45] found that NSCLC patients that over expressed ABCG2 showed resistance to platinum-based treatment, they claimed that ABCG2 might serve as a molecular target for decreasing platinum-based chemotherapeutic resistance. Taken together, we speculate that, probably, lncRNA *CASC8* rs10505477 polymorphism could affect the efficacy and toxicity of platinum-based chemotherapeutic drugs through POU5F1B-OCT-3/4-ABCG2 axis in lung cancer patients.

Finally, some deficiencies in the present study must be taken into account. First, despite that strong correlations between rs10505477 and lung cancer risk, platinum-based chemotherapy response and toxicity were observed, how this genetic variant influence *CASC8* still need further investigation. Second, only one SNP, rs10505477, was chosen in the present study, other SNPs such as rs6983267 which was linked with the SNP rs10505477 in the 8q24 region were not included. Third, all patients recruited in this study were from only two hospitals in the same city, and the number of samples was not sufficient enough.

## 5. Conclusions

In summary, for the first time, we explored the effect of *CASC8* rs10505477 polymorphism on lung cancer susceptibility, platinum-based chemotherapy response, and toxicity. Thus, the SNP rs10505477 in *CASC8* may serve as an underlying risk marker for detecting and diagnosing lung cancer, and could be used to determine the response and toxicity of platinum-based chemotherapy in lung cancer patients. However, further larger well-designed studies will be needed to clarify the biological and clinical significance of the rs10505477polymorphism and its LD with rs6983267 in lung cancer risk, platinum-based chemotherapy response, and toxicity.

## Figures and Tables

**Table 1:** Subjects characteristics.

Characteristics	Patients, *n* (%)	Controls, *n* (%)	*p*
	(*n* = 498)	(*n* = 213)	
Sex			
Male	394 (79.12)	80 (37.56)	0.00 *
Female	104 (20.88)	133 (62.44)	
Age (years)			
<50	124 (24.90)	95 (44.60)	0.00 *
≥50	374 (75.10)	118 (55.40)	
Histology			
Non-small-cell lung cancer	406 (81.53)		
Squamous-cell carcinoma	189 (37.95)		
Adenocarcinoma	217 (43.57)		
Small-cell lung cancer	69 (13.86)		
Other	23 (4.62)		
Stage (Non-small-cell lung cancer)			
I, II	13 (3.03)		
III	115 (26.81)		
IV	301 (70.16)		
Stage (Small-cell lung cancer)			
Limited	36 (52.17)		
Extensive	33 (47.83)		

Other: Mixed-cell or undifferentiated carcinoma; * *p* < 0.05.

**Table 2:** Association of chemotherapy responses with clinical pathologic features.

Clinical Pathological Features	Response to Chemotherapy		*p*
	CR + PR, *n* (%)	PD + SD, *n* (%)	
All	184 (100.00)	283 (100.00)	
Gender			
Female	28 (15.22)	68 (24.03)	
Male	156 (84.78)	215 (75.97)	0.03 *
Age(years)			
<57	89 (48.37)	145 (51.24)	0.57
≥57	95 (51.63)	138 (48.76)	
History of smoking			
Yes	123 (66.85)	163 (57.60)	0.05
No	61 (33.15)	120 (42.40)	
Histology			
Non-small-cell lung cancer	130 (70.65)	241 (85.16)	0.00 *
Squamous-cell carcinoma	76 (41.30)	91 (32.16)	
Adenocarcinoma	54 (29.35)	150 (53.00)	
Small-cell lung cancer	42 (22.83)	26 (9.19)	
Other ^a^	12 (6.52)	16 (5.65)	
Stage (Non-small-cell lung cancer)			
I, II	5 (3.42)	8 (3.11)	0.90
III, IV	141 (96.58)	249 (96.89)	
Stage (Small-cell lung cancer)			
Limited	26 (61.90)	13 (50.00)	0.45
Extensive	16 (38.10)	13 (50.00)	
Chemotherapy regimen			
Platinum/gemcitabine	82 (44.57)	110 (38.87)	0.00 *
Platinum/etoposide	43 (23.37)	25 (8.83)	
Platinum/pemetrexed	35 (19.02)	102 (36.04)	
Platinum/paclitaxel	9 (4.89)	18 (6.36)	
Platinum/docetaxel	9 (4.89)	20 (7.07)	
Other ^b^	6 (3.26)	8 (2.83)	

CR: Complete response; PR: Partial response; SD: Stable disease; PD: Progressive disease; Other ^a^: Mixed-cell or undifferentiated carcinoma; Other ^b^: Platinum/irinotecan or platinum/navelbine; * *p* < 0.05.

**Table 3:** Association between lncRNA *CASC8* rs10505477 and lung cancer risk.

Genotype	Case	Control	Dominant		Recessive		Additive	
	*n* (%)	*n* (%)	OR (95%CI)	*p*	OR (95%CI)	*p*	OR (95%CI)	*p*
GG	89 (18.39)	46 (21.90)	0.96 (0.65,1.42)	0.83	0.68 (0.44,1.06)	0.09	0.87 (0.67,1.12)	0.27
GA	243 (50.21)	101 (48.10)						
AA	152 (31.40)	63 (30.00)						

OR: Odd ratio; CI: Confidence interval.

**Table 4:** Stratification analysis of the relationship between *CASC8* rs10505477 and lung cancer risk.

Subgroup	Dominant	*p*	Recessive	*p*	Additive	*p*
	OR (95%CI)		OR (95%CI)		OR (95%CI)	
Age < 50	0.95 (0.52,1.76)	0.88	0.78 (0.36,1.68)	0.53	0.91 (0.60,1.38)	0.65
Age ≥ 50	0.96 (0.58,1.60)	0.88	0.64 (0.37,1.11)	0.11	0.84 (0.61,1.17)	0.31
Female	1.03 (0.58,1.84)	0.91	1.04 (0.53,2.06)	0.91	1.03 (0.70,1.52)	0.89
Male	0.90 (0.53,1.54)	0.70	0.51 (0.29,0.90)	0.02 *	0.76 (0.54,1.07)	0.12
Non-small-cell lung cancer	1.04 (0.70,1.56)	0.84	0.67 (0.42,1.06)	0.09	0.89 (0.68,1.16)	0.40
Squamous-cell carcinoma	0.82 (0.48,1.38)	0.45	0.75 (0.42,1.33)	0.32	0.84 (0.60,1.17)	0.30
Adenocarcinoma	1.16 (0.74,1.82)	0.51	0.52 (0.30,0.89)	0.02 *	0.87 (0.64,1.17)	0.35
Small-cell lung cancer	0.71 (0.36,1.41)	0.33	0.59 (0.28,1.26)	0.17	0.74 (0.48,1.13)	0.16
Other	0.72 (0.27,1.90)	0.50	0.73 (0.24,2.22)	0.58	0.79 (0.43,1.48)	0.46

Other: Mixed-cell, or undifferentiated carcinoma; OR: Odd ratio; CI: Confidence interval; * *p* < 0.05.

**Table 5:** Association between *CASC8* rs10505477 and chemotherapy response in lung cancers.

Genotype	CR + PR	PD + SD	Dominant		Recessive		Additive	
	*n* (%)	*n* (%)	OR (95%CI)	*p*	OR (95%CI)	*p*	OR (95%CI)	*p*
GG	36 (19.25)	49 (17.88)	1.58 (1.05,2.39)	0.03 *	0.96 (0.57,1.59)	0.86	1.22 (0.92,1.62)	0.17
GA	81 (43.32)	149 (54.38)						
AA	70 (37.43)	76 (27.74)						

CR: Complete response; PR: Partial response; SD: Stable disease; PD: Progressive disease; OR: Odd ratio; CI: Confidence interval; * *p* < 0.05.

**Table 6:** Stratification analysis of association between *CASC8* rs10505477 and response of platinum-based chemotherapy in lung cancers.

Subgroup	Dominant		Recessive		Additive	
	OR (95%CI)	*p*	OR (95%CI)	*p*	OR (95%CI)	*p*
Age < 57	1.54 (0.85,2.79)	0.15	1.00 (0.47,2.15)	0.99	1.24 (0.81,1.88)	0.32
Age ≥ 57	1.56 (0.86,2.85)	0.15	0.83 (0.40,1.69)	0.60	1.15 (0.77,1.72)	0.50
Female	1.87 (0.65,5.37)	0.25	0.45 (0.13,1.59)	0.21	1.04 (0.49,2.20)	0.92
Male	1.44 (0.91,2.26)	0.12	1.07 (0.61,1.89)	0.83	1.20 (0.88,1.64)	0.24
Non-smoke	1.75 (0.87,3.50)	0.11	0.67 (0.27,1.64)	0.38	1.18 (0.72,1.94)	0.52
Smoke	1.62 (0.95,2.75)	0.07	1.18 (0.63,2.22)	0.60	1.30 (0.92,1.86)	0.14
Non-small-cell lung cancer	1.60 (1.00,2.56)	0.05	0.97 (0.53,1.77)	0.93	1.24 (0.89,1.73)	0.20
Squamous-cell carcinoma	1.35 (0.69,2.61)	0.38	1.07 (0.50,2.33)	0.86	1.16 (0.75,1.78)	0.50
Adenocarcinoma	1.89 (0.95,3.77)	0.07	1.11 (0.40,3.05)	0.85	1.48 (0.86,2.52)	0.15
Small-cell lung cancer	1.91 (0.60,6.04)	0.27	2.01 (0.49,8.21)	0.33	1.63 (0.76,3.52)	0.21
Stage (III, IV)	1.48 (0.94,2.34)	0.09	0.87 (0.50,1.52)	0.63	1.15 (0.84,1.58)	0.37
Platinum/gemcitabine	1.45 (0.86,2.45)	0.16	0.77 (0.28,2.16)	0.62	1.60 (0.91,2.81)	0.11
Platinum/etoposide	1.46 (0.47,4.51)	0.51	1.69 (0.44,6.51)	0.45	1.37 (0.66,2.87)	0.40
Platinum/pemetrexed	1.68 (0.70,4.05)	0.25	1.06 (0.29,3.80)	0.93	1.36 (0.69,2.66)	0.37

OR: Odd ratio; CI: Confidence interval.

**Table 7:** Association between *CASC8* rs10505477 and platinum-based chemotherapy hematologic toxicity in lung cancers.

Genotype	Tolerance	Severity	Dominant		Recessive		Additive	
	*n* (%)	*n* (%)	OR (95%CI)	*p*	OR (95%CI)	*p*	OR (95%CI)	*p*
GG	61 (17.78)	19 (17.27)	1.13 (0.70,1.81)	0.63	0.93 (0.52,1.65)	0.79	1.03 (0.75,1.41)	0.86
GA	171 (49.85)	58 (52.73)						
AA	111 (32.36)	33 (30.00)						

OR: Odd ratio; CI: Confidence interval.

**Table 8:** Association between *CASC8* rs10505477 and platinum-based chemotherapy gastrointestinal toxicity in lung cancers.

Genotype	Tolerance	Severity	Dominant		Recessive		Additive	
	*n* (%)	*n* (%)	OR (95%CI)	*p*	OR (95%CI)	*p*	OR (95%CI)	*p*
GG	65 (18.47)	15 (14.85)	1.61 (0.96,2.71)	0.07	0.80 (0.42,1.50)	0.48	1.15 (0.83,1.61)	0.40
GA	168 (47.73)	61 (60.40)						
AA	119 (33.81)	25 (24.75)						

OR: Odd ratio; CI: Confidence interval.

**Table 9:** Association between *CASC8* rs10505477 and overall severe toxicity induced by platinum-based chemotherapy in lung cancers.

Genotype	Tolerance	Severity	Dominant		Recessive		Additive	
	*n* (%)	*n* (%)	OR (95%CI)	*p*	OR (95%CI)	*p*	OR (95%CI)	*p*
GG	53 (22.46)	27 (15.25)	1.22 (0.80,1.87)	0.35	0.76 (0.45,1.29)	0.31	1.01 (0.76,1.34)	0.94
GA	131 (55.51)	98 (55.37)						
AA	52 (22.03)	52 (29.38)						

OR: Odd ratio; CI: Confidence interval.

**Table 10:** Stratification analysis of relationship between *CASC8* rs10505477 and hematologic toxicity induced by platinum-based chemotherapy in lung cancers.

Subgroup	Dominant		Recessive		Additive	
	OR (95%CI)	*p*	OR (95%CI)	*p*	OR (95%CI)	*p*
Age < 57	1.05 (0.53,2.08)	0.90	0.97 (0.40,2.34)	0.94	1.01 (0.63,1.63)	0.96
Age ≥ 57	1.14 (0.57,2.25)	0.72	0.93 (0.42,2.04)	0.85	1.03 (0.66,1.60)	0.90
Female	1.01 (0.30,3.33)	0.99	0.50 (0.12,2.17)	0.36	0.80 (0.36,1.78)	0.59
Male	1.13 (0.66,1.92)	0.66	0.99 (0.52,1.90)	0.98	1.05 (0.74,1.50)	0.79
Non-smoke	1.52 (0.64,3.63)	0.35	1.00 (0.34,2.83)	0.96	1.20 (0.68,2.15)	0.53
Smoke	1.21 (0.66,2.20)	0.54	1.10 (0.55,2.19)	0.78	1.12 (0.76,1.65)	0.58
Non-small-cell lung cancer	0.59 (0.35,0.98)	0.04 *	0.45 (0.21,0.99)	0.05	0.62 (0.43,0.90)	0.01 *
Squamous-cell carcinoma	1.36 (0.63,2.91)	0.44	1.42 (0.61,3.31)	0.41	1.27 (0.78,2.05)	0.34
Adenocarcinoma	1.05 (0.46,2.40)	0.91	0.33 (0.07,1.52)	0.16	0.80 (0.44,1.47)	0.47
Small-cell lung cancer	0.92 (0.25,3.33)	0.89	0.49 (0.08,2.84)	0.43	0.80 (0.33,1.89)	0.60
Stage (III, IV)	0.98 (0.58,1.65)	0.93	0.82 (0.42,1.60)	0.57	0.93 (0.65,1.33)	0.70
Cisplatin	1.00 (0.59,1.70)	1.00	0.92 (0.48,1.75)	0.79	0.97 (0.68,1.39)	0.88
Carboplatin	1.71 (0.55,5.28)	0.35	0.93 (0.24,3.63)	0.92	1.25 (0.59,2.63)	0.56
Platinum/gemcitabine	1.04 (0.53,2.05)	0.90	1.15 (0.53,2.52)	0.72	1.07 (0.69,1.65)	0.78
Platinum/etoposide	0.85 (0.26,2.76)	0.79	0.33 (0.06,1.82)	0.20	0.70 (0.32,1.56)	0.38
Platinum/pemetrexed	1.02 (0.32,3.21)	0.98	0.22 (0.02,2.29)	0.20	0.74 (0.32,1.70)	0.48

OR: Odd ratio; CI: Confidence interval; * *p* < 0.05.

**Table 11:** Stratification analysis of relationship between *CASC8* rs10505477 and gastrointestinal toxicity induced by platinum-based chemotherapy in lung cancers.

Subgroup	Dominant		Recessive		Additive	
	OR (95%CI)	*p*	OR (95%CI)	*p*	OR (95%CI)	*p*
Age < 57	1.46 (0.75,2.85)	0.26	0.99 (0.44,2.25)	0.98	1.19 (0.76,1.86)	0.44
Age ≥ 57	2.11 (0.87,5.15)	0.10	0.63 (0.23,1.77)	0.38	1.18 (0.70,1.97)	0.54
Female	1.36 (0.48,3.83)	0.56	0.59 (0.17,2.09)	0.41	0.97 (0.49,1.92)	0.93
Male	1.57 (0.86,2.86)	0.14	0.83 (0.40,1.72)	0.62	1.15 (0.79,1.69)	0.47
Non-smoke	1.36 (0.60,3.08)	0.46	0.33 (0.09,1.21)	0.10	0.89 (0.51,1.55)	0.68
Smoke	1.80 (0.90,3.59)	0.10	1.14 (0.54,2.38)	0.73	1.32 (0.87,2.02)	0.19
Non-small-cell lung cancer	1.31 (0.72,2.38)	0.37	0.73 (0.34,1.56)	0.41	1.03 (0.70,1.53)	0.87
Squamous-cell carcinoma	1.18 (0.51,2.74)	0.70	0.91 (0.34,2.40)	0.85	1.04 (0.61,1.77)	0.89
Adenocarcinoma	1.56 (0.65,3.75)	0.32	0.48 (0.13,1.75)	0.26	1.03 (0.57,1.86)	0.92
Small-cell lung cancer	7.82 (1.36,45.07)	0.02 *	1.61 (0.28,9.07)	0.59	2.81 (0.99,7.93)	0.05
Stage (III, IV)	1.48 (0.85,2.55)	0.16	0.71 (0.36,1.42)	0.33	1.08 (0.76,1.53)	0.68
Cisplatin	1.94 (1.07,3.53)	0.03 *	0.96 (0.50,1.86)	0.91	1.30 (0.90,1.88)	0.16
Carboplatin	1.06 (0.31,3.56)	0.93	0.39 (0.04,3.42)	0.39	0.69 (0.28,1.73)	0.43
Platinum/gemcitabine	0.90 (0.42,1.91)	0.78	0.93 (0.38,2.30)	0.88	0.93 (0.57,1.54)	0.79
Platinum/etoposide	0.53 (0.15,1.88)	0.32	2.03 (0.26,15.84)	0.50	0.78 (0.28,2.15)	0.63
Platinum/pemetrexed	2.06 (0.66,6.36)	0.21	2.35 (0.71,7.80)	0.16	0.83 (0.39,1.74)	0.62

OR: Odd ratio; CI: Confidence interval;* *p* < 0.05.

**Table 12:** Stratification analysis of relationship between *CASC8* rs10505477 and overall severe toxicity induced by platinum-based chemotherapy in lung cancers.

Subgroup	Dominant		Recessive		Additive	
	OR (95%CI)	*p*	OR (95%CI)	*p*	OR (95%CI)	*p*
Age < 57	1.16 (0.64,2.12)	0.63	0.92 (0.43,1.96)	0.83	1.05 (0.69,1.58)	0.83
Age ≥ 57	1.24 (0.67,2.32)	0.49	0.67 (0.31,1.39)	0.27	0.97 (0.64,1.45)	0.87
Female	1.08 (0.38,3.04)	0.89	0.55 (0.16,1.89)	0.34	0.85 (0.42,1.70)	0.64
Male	1.26 (0.79,2.02)	0.34	0.80 (0.44,1.43)	0.45	1.04 (0.76,1.42)	0.82
Non-smoke	1.18 (0.57,2.45)	0.65	0.64 (0.25,1.67)	0.37	0.95 (0.58,1.58)	0.86
Smoke	1.19 (0.70,2.03)	0.53	0.78 (0.41,1.48)	0.45	1.00 (0.70,1.42)	1.00
Non-small-cell lung cancer	1.09 (0.67,1.76)	0.73	0.82 (0.44,1.51)	0.52	0.98 (0.71,1.36)	0.91
Squamous-cell carcinoma	1.06 (0.53,2.11)	0.87	1.02 (0.46,2.26)	0.97	1.03 (0.66,1.61)	0.9
Adenocarcinoma	1.17 (0.58,2.35)	0.66	0.47 (0.16,1.39)	0.17	0.90 (0.54,1.49)	0.67
Small-cell lung cancer	0.74 (0.20,2.73)	0.65	0.87 (0.11,6.74)	0.89	0.81 (0.30,2.21)	0.68
Stage (III, IV)	1.01 (0.64,1.59)	0.98	0.72 (0.40,1.29)	0.27	0.91 (0.66,1.25)	0.55
Cisplatin	1.19 (0.74,1.92)	0.46	0.81 (0.46,1.44)	0.47	1.01 (0.74,1.39)	0.93
Carboplatin	1.46 (0.53,4.02)	0.46	0.61 (0.16,2.37)	0.48	1.05 (0.52,2.10)	0.89
Platinum/gemcitabine	0.80 (0.43,1.50)	0.49	0.90 (0.42,1.90)	0.77	0.88 (0.58,1.33)	0.54
Platinum/etoposide	0.65 (0.19,2.17)	0.48	0.84 (0.13,5.57)	0.86	0.75 (0.30,1.88)	0.54
Platinum/pemetrexed	1.25 (0.50,3.12)	0.64	0.75 (0.39,1.37)	0.41	0.72 (0.37,1.40)	0.33

OR: Odd ratio; CI: Confidence interval.
